# Prediction of posttraumatic stress disorder among adults in flood district

**DOI:** 10.1186/1471-2458-10-207

**Published:** 2010-04-26

**Authors:** Peng Huang, Hongzhuan Tan, Aizhong Liu, Shuidong Feng, Mengshi Chen

**Affiliations:** 1 School of Public Health, Central South University, Xiangya Road 110, Changsha, Hunan 410078, PR China; 2 School of Public Health, Nanchang University, Bayi Road 603, Nanchang, Jiangxi 330006, PR China; 3 School of Public Health, University of South China, Changsheng Road 28, Hengyang, Hunan 421001, PR China

## Abstract

**Background:**

Flood is one of the most common and severe forms of natural disasters. Posttraumatic stress disorder (PTSD) is a common disorder among victims of various disasters including flood. Early prediction for PTSD could benefit the prevention and treatment of PTSD. This study aimed to establish a prediction model for the occurrence of PTSD among adults in flood districts.

**Methods:**

A cross-sectional survey was carried out in 2000 among individuals who were affected by the 1998 floods in Hunan, China. Multi-stage sampling was used to select subjects from the flood-affected areas. Data was collected through face-to-face interviews using a questionnaire. PTSD was diagnosed according to DSM-IV criteria. Study subjects were randomly divided into two groups: group 1 was used to establish the prediction model and group 2 was used to validate the model. We first used the logistic regression analysis to select predictive variables and then established a risk score predictive model. The validity of model was evaluated by using the model in group 2 and in all subjects. The area under the receiver operation characteristic (ROC) curve was calculated to evaluate the accuracy of the prediction model.

**Results:**

A total of 2336 (9.2%) subjects were diagnosed as probable PTSD-positive individuals among a total of 25,478 study subjects. Seven independent predictive factors (age, gender, education, type of flood, severity of flood, flood experience, and the mental status before flood) were identified as key variables in a risk score model. The area under the ROC curve for the model was 0.853 in the validation data. The sensitivity, specificity, positive predictive value (PPV) and negative predictive value (NPV) of this risk score model were 84.0%, 72.2%, 23.4%, and 97.8%, respectively, at a cut-off value of 67.5 in the validation data.

**Conclusions:**

A simple risk score model can be used to predict PTSD among victims of flood.

## Background

Flood is one of the most common and severe forms of natural disasters. It can result in direct economic and property losses, physical injuries, deaths, and psychological injuries. Posttraumatic stress disorder (PTSD) is a common disorder among victims of various disasters such as traffic accidents[1,2], violent crimes[3], hurricanes[4], earthquakes[5,6], and floods [7-10]. PTSD is also a severe and complex disorder precipitated by exposure to psychologically distressing events, and it is characterized by persistent intrusive memories about the traumatic event, persistent avoidance of stimuli associated with the trauma, and persistent symptoms of increased arousal[11].

Floods occur frequently in China. A severe flood that struck China's Hunan province in 1998 left hundreds of thousands of residents homeless, and damaged many infrastructural and agricultural projects. It is of great importance to find ways of promptly identifying flood victims who are likely to develop PTSD to enable the government take timely measures to protect the health of such victims. Currently, there are no PTSD prediction models that can be applied to flood victims. The aim of this study was therefore to identify determinants of PTSD and to develop a risk score model to predict PTSD among flood victims.

## Methods

### Study area and population

The 1998 floods in China affected over 180 million people. It is estimated that the flood displaced 18.393 million people; destroyed 6.85 million houses; caused 4,150 deaths; and yielded a direct economic loss of about $32 billion (New Report from Ministry of Health, China, 1999). Hunan was the most severely affected province. Victims who had been directly exposed to the 1998 flood in Hunan formed our target population. The study area covered the catchment area of the Dongting Lake (north of Hunan) and the west of Hunan.

The catchment area of the Dongting Lake is located south of the middle reaches of the Yangzi River in southern China. It is usually warm, humid, and rainy during summer. The area, which is flood-prone, experienced soaked and collapsed floods in 1998. It consists of 31 counties; covers an area of 31,000 km^2^; and has an estimated human population of 11.3 million. Residents who live in this area share similar natural conditions and socio-economic and health status. The majority of them are farmers with low levels of education. The area within the west of Hunan covers 7 counties affected by the flash floods of 1998. These counties also share similar socio-demographic characteristics.

We used a multi-stage stratified and cluster sampling method to select study subjects. Firstly, we randomly selected 7 counties from 31 counties that suffered soaked and collapsed floods (Yueyang, Lingxiang, Huarong, Qianlianghu, Ziyang, Anxiang, Datonghu) and 1 county from 7 counties that experienced flash flood (Longshan). Then, by a systematic sampling approach, we randomly sampled 50% of townships in the selected counties, 50% of villages in the selected townships, and 50% of households in the selected villages. All family members in the selected households aged 16 years and older; experienced the flood; and willing to be interviewed were invited to participate in our study.

### Flood type and severity

Flood was classified into 3 types: soaked flood, collapsed embankment flood, and flash flood. Soaked floods are also called drainage-problem floods, occurred as a result of regular drainage systems not able to handle high precipitation levels. Collapsed embankment floods, which are also called river flood, are caused by flooding of the river outside its regular boundaries, often as a result of high precipitation levels. Flash floods usually occur as a result of local rainfalls with high intensity[12].

The severity of flood suffered was also divided into 3 categories: mild (affected area <50%), intermediate (affected area 50%-75%), and severe (affected area >75%), according to the standard setup by the Chinese flood management authority.

### Data collection

The survey was conducted between January and May 2000. 40 trained interviewers, who worked at the local Centres for Disease Control and Prevention and had a bachelor's degree or higher, carried out face-to-face interviews using a questionnaire to obtain demographic data, to ascertain PTSD, and to measure personality and psychological characteristics. The interviewers received on-site supervision from psychologists. The project was approved by the Research Ethics Board of Central South University, and all subjects agreed to participate in the investigation. All interviews occurred in the study subjects own home, in a private room with no other person present. Interviews lasted for about 20 minutes. To facilitate the study, we contacted the study subject by telephone before the interview. If the scheduled time was not convenient for him/her, we changed it.

The diagnosis of PTSD was made according to the Diagnostic and Statistical Manual of Mental Disorders, Fourth Edition (DSM-IV) criteria[11], which included 17 symptoms scored as 0 = none, 1 = slight, 2 = moderate, 3 = severe, and 4 = extreme. Symptoms with scores = 2 were defined as positive. The 17 symptoms of PTSD were further divided into 3 groups, representing 3 diagnostic criteria B, C, and D. Criterion B symptoms represented the re-experiencing cluster, and subjects were defined as B symptom positive if they showed one or more positive items in the B group. Criterion C symptoms represented the avoidance cluster, and subjects were defined as C symptom positive if they showed 3 or more positive items in the C group. Criterion D symptoms represented the hyperarousal cluster, and subjects were defined as D symptom positive if they showed two or more positive items in the D group. In addition, there were criteria A and E for the diagnosis of PTSD. Criterion A represented exposure to an extreme traumatic stressor involving direct personal experience of an event, witnessing an event, learning about unexpected or violent death, serious harm, or threat of death or injury experienced by a family member or another close associate (A1); and the person's response to the event must involve intense fear, helplessness, or horror (A2). All subjects in our study witnessed the 1998 flood and experienced the threat of death or injury from the flood. Also, all the probable PTSD-positive subjects met the A2 criterion. Criterion E represented the disturbance lasting more than 1 month. Subjects were diagnosed as having PTSD if Criteria A, B, C, D, and E symptoms were all positive. We assessed all symptoms, including the time and duration of occurrence. The questionnaire for PTSD had been tested in Chinese populations and had been proved to be valid and reproducible [8].

All interviewers participated in a 2-day training program, which focused on the questionnaires. A working manual was provided to ensure that all interviewers had the same understanding for the questionnaire. The completed questionnaires were checked by the coordinator (one coordinator in each county) of the study. If a questionnaire was found to be incomplete or inconsistent, the interview was repeated for the same subject to reduce missing data as much as possible.

### Statistical analysis

We randomly divided the data sets into two groups, one group (group 1: approximately 70% of the samples) for the creation of the prediction model and the other group (group 2: approximately 30% of the samples) for the validation of the prediction model.

We first used stepwise forward logistic regression analysis to select the predictive variables. The dependent variable was PTSD (yes = 1, no = 0). Based on professional judgement and literature [13-16], we selected the following potential predicting variables into the initial regression model: age (x_1_,16~ = 1; 35~ = 2; 55~ = 3), gender (x_2_, male = 0; female = 1), education (x_3_, illiterate = 3; elementary school = 2; high school or higher = 1), occupation (x_4_, farmer = 1; nonfarmer = 0), type of flood,(x_5_, soaked = 1; collapsed = 2; flash flood = 3), severity of flood (X_6_, mild = 1; moderate = 2; severe = 3), flood experience (X_7 _= X_7.1_+ X_7.2_+ X_7.3_+ X_7.4_+ X_7.5_+ X_7.6. _X_7.1_: were you trapped and waited for rescue during the flood, yes = 1, no = 0; X_7.2_: were you seriously injured during the flood, yes = 1, no = 0; X_7.3_: were your relatives or friends seriously injured during the flood, yes = 1, no = 0; X_7.4_: did you witness others drowning during the flood, yes = 1, no = 0; X_7.5_: was this flood your first experience of floods, yes = 1, no = 0; X_7.6_: was your house damaged by the flood, yes = 1, no = 0), and mental status before flood (X_8 _= X_8.1_+ X_8.2_+ X_8.3_+ X_8.4_. X_8.1_: would you consider yourself tensed or highly strung-up, yes = 1, no = 0; X_8.2_: do you often feel life is very boring, yes = 1, no = 0; X_8.3_: do you often feel lonely, yes = 1, no = 0; X_8.4_: are you easily hurt when people find faults with you or your work, yes = 1, no = 0). All potential predicting variables were valued according to the levels of PTSD prevalence.

To develop a simple risk score, the risk factors identified through multivariate logistic regression were assigned an integer coefficient. Integers were chosen to be approximately proportional to the estimated continuous coefficients from the logistic model. Assignment of points to risk factors was based on a linear transformation of the corresponding β regression coefficient. The coefficient of each variable was divided by the lowest β value and rounded to the nearest integer[17]. The final value of risk score predictive model was the sum of the risk scores mentioned above. Group 2 was used to confirm the accuracy of the risk score model by calculating the area under ROC curve. We then assessed the sensitivity, specificity, crude agreement (CA), positive predictive value (PPV) and negative predictive value (NPV) of the risk score model at different cut-off values for subjects in group 2 and for all subjects, using the diagnostic result of DSM-IV criteria as the gold standard. The CA was obtained by the sum of true positive and true negative divided by total number of subjects. The CA assumed that a prediction model had no diagnostic value if CA = 0, and that a model was invariably correct if CA = 1. SPSS 13.0 was used for all the data analysis.

## Results

A total of 8 counties, 40 towns, 310 villages, 13,450 households, and 29,285 individuals aged 16 years and older were selected for the study. Among the 29,285 subjects 1,128 (3.9%) refused to participate, 1,035 (3.5%) had not been interviewed, 1,644 (5.6%) had incomplete data, and 25,478 had complete data, yielding a response rate of 87.0%. A total of 2,336 subjects were probable PTSD positive, yielding a probable positive rate of 9.2%. For the 25,478 subjects in the final analysis, 17,846 (70%) were randomly selected to group 1 and 7,632 (30%) to group 2. There was no significant difference in baseline characteristics and probable PTSD rates (P > 0.05) between the two study groups (Table 1).

**Table 1:** Sample distribution and probable PTSD-positive rates in 2 groups

Variables	Group 1 (N = 17846)		Group 2 (N = 7632)		Total (N = 25478)		
	
	N	%	N	%	N	%	Probable positive rate of PTSD (%)
x_1 _age							
16~	8025	45.0	3364	44.1	11389	44.7	8.4
35~	7019	39.3	3046	39.9	10065	39.5	9.2
55~	2802	15.7	1222	16.0	4024	15.8	11.3
x_2 _gender							
male	8441	47.3	3557	46.6	11998	47.1	8.2
female	9405	52.7	4075	53.4	13480	52.9	10.2
x_3 _education							
high school or higher	1749	9.8	733	9.6	2482	9.7	3.1
elementary school	14360	80.5	6168	80.8	20528	80.6	8.0
illiterate	1737	9.7	731	9.6	2468	9.7	24.9
x_4 _occupation							
nonfarmer	1534	8.6	678	8.9	2212	8.7	6.2
farmer	16312	91.4	6954	91.1	23266	91.3	9.4
x_5 _type of flood							
soaked	9166	51.4	3936	51.6	13102	51.4	2.9
collapsed	6527	36.6	2799	36.7	9326	36.6	12.9
flash flood	2153	12.1	897	11.8	3050	12.0	24.9
x_6 _severity of flood							
mild	7984	44.7	3384	44.3	11368	44.6	1.7
moderate	3965	22.2	1720	22.5	5685	22.3	13.9
severe	5897	33.0	2528	33.1	8425	33.1	16.1
x_7.1 _were you trapped and waited for rescue during the flood?							
no	17364	97.3	7437	97.4	24801	97.3	8.5
yes	482	2.7	195	2.6	677	2.7	32.2
x_7.2 _were you seriously injured during the flood?							
no	17717	99.3	7585	99.4	25302	99.3	9.0
yes	129	0.7	47	0.6	176	0.7	32.4
x_7.3 _were your relatives or friends seriously injured during the flood?							
no	17654	98.9	7547	98.9	25201	98.9	9.0
yes	192	1.1	85	1.1	277	1.1	28.5
x_7.4 _did you witness others drowning during the flood?							
no	17753	99.5	7579	99.3	25332	99.4	9.0
yes	93	0.5	53	0.7	146	0.6	33.6
x_7.5 _was this flood your first experience of floods?							
no	7440	41.7	3169	41.5	10609	41.6	2.7
yes	10406	58.3	4463	58.5	14869	58.4	13.8
x_7.6 _was your house damaged by the flood?							
no	12109	67.9	5243	68.7	17352	68.1	4.4
yes	5737	32.1	2389	31.3	8126	31.9	19.3
x_8.1 _would you consider yourself tensed or highly strung-up?							
no	15078	84.5	6406	83.9	21484	84.3	7.5
yes	2768	15.5	1226	16.1	3994	15.7	18.1
x_8.2 _do you often feel life is very boring?							
no	15766	88.3	6707	87.9	22473	88.2	7.6
yes	2080	11.7	925	12.1	3005	11.8	20.7
x_8.3 _do you often feel lonely?							
no	16217	90.9	6901	90.4	23118	90.7	7.8
yes	1629	9.1	731	9.6	2360	9.3	22.6
x_8.4 _are you easily hurt when people find faults with you or your work?							
No	15081	84.5	6428	84.2	21509	84.4	7.2
Yes	2765	15.5	1204	15.8	3969	15.6	19.8
y PTSD							
No	16209	90.8	6933	90.8	23142	90.8	
Yes	1637	9.2	699	9.2	2336	9.2	

Note: Chi-square tests of all variables showed no significant differences between the two groups (P > 0.05).

Table 2 shows results of the stepwise logistic regression analysis among group 1 subjects. There were 7 variables entered into the prediction model, namely, age (X_1_), gender (X_2_), education (X_3_), type of flood (x_5_), severity of flood (X_6_), flood experience (X_7_), and mental status before flood (X_8_). The Logistic probability model was as follows:

**Table 2:** Significant PTSD predictive variables included in the logistic model

				95.0% C.I. for OR		
					
Variable	β	Sig.	Odds ratio	Lower	Upper	risk score (points)
x_1_(age)	0.085	0.031	1.089	1.008	1.176	1
X_2_(gender)	0.213	0.000	1.237	1.104	1.386	3
X_3_(education)	1.088	0.000	2.970	2.625	3.360	13
X_5_(type of flood)	0.689	0.000	1.991	1.811	2.189	8
X_6_(Severity of flood)	0.327	0.000	1.387	1.275	1.510	4
X_7_(Flood experience)	0.672	0.000	1.957	1.807	2.120	8
X_8_(mental status)	0.519	0.000	1.680	1.601	1.763	6
Constant	-8.091	0.000	0.000			

**Figure 1:**
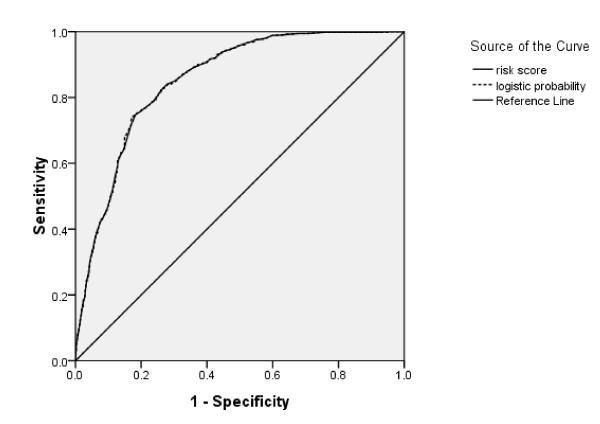
**The ROC curve of logistic probability model and risk score model for group 2 subjects**.

Based on β regression coefficient of each variable, risk score was calculated with Singh' method[17]. The final risk score model is as follow:

For example, if a subject is 38 years old (X_1 _= 2); male (X_2 _= 0); illiterate(X_3 _= 3); experienced moderate flood (X_6 _= 2); suffered flash flood(X_5 _= 3); and with the scores of 2 and 3 for flood experience (X_7_)and mental status before flood (X_8_) respectively, his total risk score will be as follows:

The area under ROC curve for both the logistic probability model and the risk score model for group 2 were 0.853 (Figure 1). This means that the risk score model, which is much simpler and easier to use, could yield similar results as the logistic probability model.

Table 3 compares the validity of the risk score model under different cut-off values (based on individual total risk scores) in different groups. It appears risk score 63.5 ~ 69.5 may be an acceptable cut-off value yielding a sensitivity of 80.5% ~ 89.4%, specificity of 63.4% ~ 75.0%, CA of 65.8% ~ 75.5%, PPV of 19.8% ~ 24.5%, and NPV of 97.4% ~ 98.3% in group 2 (Table 3).

**Table 3:** The validity of predictive model under different cut-off value in different populations (%)

	Group 2					All subjects				
		
Cutoff value	Sen	Spe	CA	PPV	NPV	Sen	Spe	CA	PPV	NPV
25.0	100.0	0.0	9.2	9.2		100.0	0.0	9.2	9.2	
39.5	99.9	8.2	16.6	9.9	99.9	99.9	8.4	16.8	9.9	99.9
57.5	95.3	51.2	55.2	16.5	99.1	94.6	51.4	55.4	16.4	99.0
62.5	90.6	60.4	63.2	18.7	98.5	90.3	60.6	63.3	18.8	98.4
63.5	89.4	63.4	65.8	19.8	98.3	88.8	63.3	65.6	19.6	98.2
64.5	88.0	65.9	67.9	20.6	98.2	87.4	65.8	67.8	20.5	98.1
65.5	86.8	67.4	69.2	21.2	98.1	86.5	67.3	69.1	21.1	98.0
66.5	85.1	69.6	71.0	22.0	97.9	85.3	69.4	70.9	22.0	97.9
67.5	84.0	72.2	73.3	23.4	97.8	83.3	71.8	72.9	23.0	97.7
68.5	82.3	73.9	74.7	24.1	97.6	81.5	73.4	74.1	23.6	97.5
69.5	80.5	75.0	75.5	24.5	97.4	80.2	74.5	75.0	24.1	97.4
70.5	78.8	76.4	76.6	25.2	97.3	78.8	75.9	76.2	24.8	97.3
71.5	76.4	79.4	79.1	27.2	97.1	76.0	79.0	78.7	26.8	97.0
73.5	71.1	83.3	82.2	30.0	96.6	69.9	83.0	81.8	29.3	96.5
78.5	49.6	89.3	85.7	31.9	94.6	51.8	89.6	86.1	33.5	94.8
97.5	10.9	98.8	90.7	47.8	91.7	11.6	98.8	90.8	49.4	91.7
130.0	0.0	100.0	90.8		90.8	0.0	100.0	90.8		90.8

Based on individual risk scores calculated from our risk score model, we can predict the probability of PTSD occurrence. For the case mentioned above (risk score = 107), if 65 is selected as cut-off value, we will consider this individual to be at a high risk of developing PTSD. The higher the risk score is, the greater the probability of the individual developing PTSD.

## Discussion

PTSD is a common psychological disorder in disaster-affected populations. It has been widely used to evaluate the psychological impact of natural disasters and accidents[1,2,6-10]. To our knowledge this is the first study to explore the prediction of PTSD by risk score model among flood victims in a large population. The method of risk score has been widely used for prediction or screening of disease because of its simplicity and ease of interpretation [17-21]. In our study, a risk score model was established according to β regression coefficient from logistic regression analysis, which included 7 predictive variables. These variables included demographic characteristics (x_1_, x_2_, x_3_), type of flood (x_5_), severity of flood (x_6_), flood experience(x_7_) and mental status before flood (x_8_). In order to make the prediction model simpler and easier to understand, we combined X_7.1_-X_7.6 _into X_7 _and X_8.1_-X_8.4 _into X_8_, with the cumulative score as their score. The area under ROC curve for the logistic probability and risk score models were very similar but since the risk score model is much simpler and easier to use, we recommend its use in PTSD prediction among flood victims. The suitability of the risk score model is further supported by a sensitivity, specificity, positive predictive value (PPV) and negative predictive value (NPV) of 84.0%, 72.2%, 23.4%, and 97.8%, respectively at the cut-off value of 67.5 in group 2.

To make the model have better predictive value, we used 4 mental status-related variables (x_8.1_-x_8.4_) as potential predictors representing one's mental status before flood, in addition to age, gender, education, and severity of flood suffered, which were important predictors from previous studies [13,14,16]. Although there have been different findings about the strength of association between mental health status before trauma and PTSD [13,14,16], coupled with the fact that retrospective reports may be influenced by current symptoms, our study results still yielded valuable information. Mental status prior to flood was an effective predictor of PTSD in our study.

In view of the fact that our study focused on the prediction of PTSD, rather than screening, only demographic characteristic(x_1_, x_2_, x_3_), type of flood (x_5_), severity of flood (x_6_), flood experience(x_7_) and mental status before flood (x_8_) were included in our predictive model(early symptoms of PTSD were not included). We listed sensitivity, specificity, PPV, and NPV at different cut-off values so allow public health workers choose the appropriate cut-off value for their PTSD predictions. For example, if the goal is to find as many PTSD cases as possible, one could use 39.5 as the cut-off value, and this will raise the sensitivity level to 99.9%. If the goal is to reduce the false positives, one could use 97.5 as the cut-off value to yield a specificity level of 98.8%.

Our PTSD prediction model was validated with a separate sample, which showed its true and reliable performance when applied on other populations. All the predictive variables included in the model could be easily obtained through a simple questionnaire after a flood. Compared with other PTSD screening models, which included some PTSD early symptoms as predictors [13-16,22], our model showed lower sensitivity and specificity. However, it could be used to predict the possible occurrence of PTSD immediately after a flood. Our model, therefore, is significant in public health programs.

Our study used a retrospective survey method to investigate the impacts of flood. As a result, recall bias and information bias could occur. However, because interviewers did not know who had PTSD or who had not at the time of interviewing, the recall bias and information bias, if any, may have occurred randomly.

Another limitation of our study is the fact that the diagnosis of PTSD was made using a questionnaire administered by interviewers. Although the interviewers received on-site supervision from psychologists, the diagnosis of PTSD may not be accurate. In view of this, all suspected cases were diagnosed as 'probable PTSD'.

The potential predictive variable considered in our model was selected based on professional judgement as well as literature. Other variables, such as economic loss, property damage, and family history of mental illness were considered in some preliminary analyses of our data. However, since those variables were not found to be statistical significant in univariate analyses, we did not include them in the multivariable logistic regression analysis. Although our model has not been proved to be an optimal one, it is a practical and useful model for PTSD prediction at least for now. Its performance in other populations needs to be further investigated.

## Conclusions

The risk score based predictive model for PTSD developed in this study has an acceptable predictive value with favourable applicability, and can be used to identify persons at risk of PTSD during floods.

## List of abbreviations

PTSD: Posttraumatic stress disorder; CA: crude agreement; PPV: positive predictive value; NPV: negative predictive value; ROC: receiver operating curve.

## Competing interests

The authors declare that they have no competing interests.

## Authors' contributions

PH conceived of the manuscript idea, drafted the manuscript and performed the statistical analysis. HT provided supervision and guidance in the design, analysis and writing of the manuscript. AL, SF and MC participated in the design and coordination of the original study. All authors read and approved the final manuscript.

## Pre-publication history

The pre-publication history for this paper can be accessed here:

http://www.biomedcentral.com/1471-2458/10/207/prepub
